# Risk factors for cognitive decline in type 2 diabetes mellitus patients in Brazil: a prospective observational study

**DOI:** 10.1186/s13098-022-00872-3

**Published:** 2022-07-27

**Authors:** Ana Cristina Ravazzani de Almeida Faria, Joceline Franco Dall’Agnol, Aline Maciel Gouveia, Clara Inácio de Paiva, Victoria Chechetto Segalla, Cristina Pellegrino Baena

**Affiliations:** 1 Postgraduate Program in Health Sciences, Pontifícia Universidade Católica do Paraná (PUCPR), Rua Imaculada Conceição, 1155, Curitiba, 80215-901 Brazil; 2 School of Medicine, Pontifícia Universidade Católica do Paraná (PUCPR), Curitiba, Paraná Brazil

**Keywords:** Cognitive dysfunction, Type 2 Diabetes Mellitus, Risk factors

## Abstract

**Background:**

Type 2 Diabetes Mellitus (T2DM) patients are twice as likely to develop dementia. The study’s goal was to evaluate cognitive performance and risk factors for cognitive decline in this population.

**Methods:**

Prospective observational study was conducted with 400 T2DM adults, of whom, during routine baseline and follow-up appointments, had socio-demographic, clinical, and laboratory data collected, and underwent physical examination, screening for depression symptoms (Patient Health Questionaire-9-PHQ-9), and cognitive tests: Mini-Mental State Examination (MMSE), Semantic Verbal Fluency Test, Trail Making Test A/B, and Word Memory Tests. Each cognitive test score was converted to a z-score and its average resulted in a new variable called Global Cognitive z-Score [GCS(z)]. Averages of the cognitive test scores and GCS(z) at both moments were compared by the Student’s T-Test for paired samples. Multivariate binary logistic regression models were built to assess the association of GCS(z) < zero with risk factors for cognitive decline at the baseline and follow-up.

**Results:**

After exclusions, 251 patients were eligible, being 56.6% female, mean age of 61.1 (± 9.8) years, 12.6 (± 8.9) years of DM duration, and 7.6 (± 4.2) years of school education. Follow-up had 134 patients reevaluated and took place after a mean of 18.4(± 5.0) months. Eleven (14%) patients with a GCS(z) ≥ 0 at baseline turned into a GCS(z) < 0 at follow-up. There were no significant differences between the means of cognitive test scores and GCS(z) at the two evaluation moments. At the baseline, the multivariate logistic regression model identified five risk factors associated with GCS(z) < zero: age ≥ 65 years, schooling ≤ 6 years, arterial hypertension, depression symptoms, and diabetic retinopathy (DR), with odds ratio (OR) and 95% confidence interval (CI95%) respectively: 5.46 (2.42–12.34); 12.19 (5.62–26.46); 2.55 (0.88–7.39); 3.53 (1.55–8.07) e 2.50 (1.18–5.34). At follow-up, the risk factors for GCS(z) < zero were: schooling ≤ 6 years, DM duration ≥ 10 years, depression symptoms, arterial hypertension, and cardiovascular disease (CVD), OR and CI95% respectively: 10.15 (3.68–28.01); 2.68 (0.96–7.48); 4.92 (1.77–13.70); 7.21 (1.38–35.71) e 5.76 (1.93–17.18).

**Conclusions:**

Based on our results, cognitive evaluation and follow-up should be incorporated on the routine of T2DM patients, especially for those with advanced age, low education level, prolonged DM duration, arterial hypertension, depression symptoms, CVD, and DR.

**Supplementary Information:**

The online version contains supplementary material available at 10.1186/s13098-022-00872-3.

## Background

According to The Global Burden of Disease Study*,* the number of people living with dementia in 2016 was 43.8 million [1], and it is estimated that in 2030 there will be 74 million, reaching 131 million in 2050 [2]. However, the most striking is that 58% of these people live in countries classified as emerging economies, with an even greater projection of growth for the next few years [3]. Following the global trend, there was an increase in dementia incidence/100,000 inhabitants of 4.5% and prevalence/100,000 inhabitants of 7.8%, in recent decades in Brazil [4].

Chronic non-communicable diseases are responsible for a high number of deaths, in addition to the loss of quality of life and significant financial impact on public health [5]. Among these diseases, Type 2 Diabetes Mellitus (DM) affected about 463 million adults worldwide in 2019, with an expected increase to up to 642 million in 2040, according to the International Diabetes Federation. In Latin America, Brazil is the country with the highest number of people with DM and the fifth in the world, which means 16.8 million people, being important to highlight that one in five of these people is over 65 years old [6].

Longitudinal studies identified that the risk for dementia is greater in the population with DM [7–14]. The risk is 1.73 for all types of dementia, 1.53 for Alzheimer’s Dementia (AD), and 2.27 for vascular dementia, when compared to people without DM [13, 15]. Furthermore, epidemiological studies also indicate that 80% of patients with AD have DM or glucose intolerance [16]. Subtle changes in cognition are seen in DM patients in all age groups, but minimal cognitive dysfunction (MCD) and dementia become more evident after 60 years of age [13].

Even MCD that does not interfere with self-care can progress and affect executive functions that involve solving everyday problems, change in habits, and judgment when faced with new situations [17]. All of this can interfere with self-care and quality of life, as well as make the patient dependent, burdening families and communities.

The study of the potential impact of controlling seven risk factors for dementia [DM, arterial hypertension (AH), obesity, smoking, depression, low education, and sedentary lifestyle] in reducing its prevalence, identified that, together, they contribute to almost half of the AD cases globally (17.2 million). It is estimated that a 10–25% reduction in these risk factors can potentially prevent up to 1.1–3.0 million cases of AD worldwide [10].

Given the high prevalence of people with T2DM and the aging population growth, with a consequent increase in dementia rates, this study aimed to assess the cognitive alterations and their risk factors in a population of patients with T2DM in an emerging economy country, making possible the implementation of prevention, detection, and future treatment strategies.

Considering the hypothesis that a cognitive decline over time may occur in the T2DM population and that there may be modifiable and non-modifiable exposure factors among individuals at higher risk for this decline, the main study objective was to evaluate the cognitive performance and decline in 12–24 months and secondary objective was to identify demographic, clinical and laboratory exposure factors for cognitive decline on this population.

## Materials and methods

### Study design and sample

A longitudinal prospective study was conducted from September 2017 to December 2020, in a tertiary hospital in southern Brazil. In our convenience sample, patients were sequentially recruited, according to their attendance at their routine appointments. They were evaluated upon their arrival at the clinic at baseline and reassessed within at least 12 and no later than 24 months after baseline [18]. Patients over 18 years of age with T2DM of both genders were included. Patients with T2DM were considered those who did not need insulin in the first 3 years of the disease and had no history of ketonuria or ketonemia at diagnosis [19]. Four hundred patients were initially evaluated.

Patients with a history of using medicines that alters cognition (benzodiazepines, hypnotics, antipsychotics, tricyclic antidepressants, anticonvulsants, anticholinergics, and antihistamines) were excluded, as well as those who were unable to perform the cognitive tests due to illiteracy, vision or hearing impairment. Those with a previous diagnosis of dementia of any etiology, stroke, traumatic brain injury, Parkinson’s disease, schizophrenia, or any other situation that affects cognition were also excluded. Patients that met dementia criteria at MMSE at initial evaluation were excluded as well.

The study was approved by the Research Ethics Committee of Pontificia Universiade Católica do Paraná and conducted following the principles of the Declaration of Helsinki [20].

### Data collection

Participants answered a questionnaire containing demographic data (age, gender, race, marital status, and education), lifestyle habits (physical activity, alcohol consumption, smoking), and medical history [DM onset age, acute complications such as severe and chronic hypoglycemia such as retinopathy, neuropathy, and DM kidney disease, cardiovascular disease (CVD)], in addition to comorbidities and medications used. Data collected were age (years), auto-referred gender (male/female/other), auto- referred race (white, black, brown, or yellow), marital status (single, married or stable union, separated or divorced), education (years of formal school), lifestyle habits (physical activity, alcohol, and tobacco consumption).

The data from physical activity was collected as the type of exercise (aerobic/resistance), the number of days/week, and minutes/day. It was considered physically active participants who met the criteria of at least 150 min of moderate or 75 min of intensive aerobic exercise per week. Tobacco and alcohol consumption was measured as former use or past/never used.

Medical history data were retrieved from medical records and collected directedly by anamnesis upon baseline. Data collected were: T2DM onset date, last year’s severe hypoglycemia episodes, chronic complications (retinopathy, neuropathy, and DM kidney disease), cardiovascular disease (CVD), other comorbidities, and all current medications used for diabetes and comorbidities. Severe hypoglycemia was defined as that in which the patient needed help from others for treatment and/or had a reduced level of consciousness, with an improvement of symptoms after treatment [19, 21].

The following data was collected from physical examination: Body Mass Index (BMI) (kg/m^2^), Abdominal and Neck Circumference (cm), Systolic Blood Pressure (SBP) (mmHg), and Diastolic Blood Pressure (DBP) (mmHg). Arterial hypertension diagnosis was defined as two measurements of SAP ≥ 140 mmHg and/or DBP ≥ 90 mmHg or yet if using antihypertensive medication [22].

Retina clinical examination was performed by the ophthalmologists by retinal mapping under drug-induced mydriasis by indirect binocular ophthalmoscopy and slit-lamp biomicroscopy and, when indicated, by fluorescein angiography and optical coherence tomography classified as an absence of diabetic retinopathy (DR), non-proliferative DR, proliferative DR, and macular edema [19, 23, 24].

Diabetic neuropathy was considered in the presence of clinical symptoms and signs compatible with peripheral sensory-motor neuropathy according to the criteria recommended by the Guidelines of the Brazilian Diabetes Society, based on peripheral neurological clinical examination [19].

DM kidney disease was considered in the presence of eGFR < 60 ml/min/1.73m^2^ and/or ACR > 30 mg/g and/or persistently elevated creatinine for more than 3 months, following the recommendations of KDIGO (Kidney Disease: Improving Global Outcomes) and the guidelines of the Brazilian Diabetes Society [19, 25].

Laboratory test data were retrieved from the hospital’s laboratory test files within a period of up to 3 months before the cognitive evaluation. Data collect were: glycated hemoglobin (HBA1c) (%), fasting glucose (mg/dl), total cholesterol (mg/dl), low-density lipoprotein (LDL) (mg/dl), high-density lipoprotein cholesterol (HDL) (mg/dl), triglycerides (mg/dl), thyroid-stimulating hormone (mUI/L), free thyroxine (ng/dl), B12 vitamin (pg/ml), creatinine (mg/dl), urea (mg/dl) and urinary albumin-to-creatinine ratio (ACR)(mg/g creatinine). Creatinine values were used to calculate the estimated glomerular filtration rate (eGFR) adjusted for age and gender using the CKD-EPI (Chronic Kidney Disease Epidemiology Collaboration) formula [25–27].

Diagnosis of dyslipidemia was based on the Guidelines of the Brazilian Cardiology Society (LDL ≥ 100 mg/dl if intermediate cardiovascular risk, ≥ 70 mg/dl if high cardiovascular risk, and ≥ 50 mg/dl if very high cardiovascular risk; and/or HDL ≤ 45 mg/dl and/or Triglycerides ≥ 150 mg/dl) and/or if being treated with lipid-lowering medication [28].

In the follow-up assessment, the patients again answered the lifestyle habits, medical history, and depression symptoms questionnaire, as well as underwent the same tests to assess their cognitive function.

### Cognitive and depression symptoms assessment

The cognitive tests performed, validated in Portuguese, were the Trail Making Test A and B to assess sustained attention, mental flexibility, executive function, spatial/visual organization, and processing speed [29–32], and the semantic Verbal Fluency test to assess semantic memory storage capacity, ability, ability to retrieve information from memory, and processing of executive functions [31–34]; the CERAD (The Consortium to Establish a Registry for Alzheimer’s Disease) Word List Test to assess memory [32–34]; and the MMSE, to screen patients at risk of being diagnosed with dementia and make a global assessment of cognition, covering aspects of orientation, memory, attention, calculation, language, and comprehension [32, 35–41]. The cutoff value of MMSE varies according to education level, and scores with values below the cutoff corrected by the education level in the Brazilian population were used to indicate dementia risk [39]. (Additional file 1).

The PHQ-9 (Patient Health Questionaire-9), validated in Portuguese, was carried out and values above 9 were considered as a risk of a diagnosis of major depression [42, 43]. (Additional file 2).

To avoid bias, the investigators who applied the tests were previously trained by the same psychologist and authorized to apply the tests after being certificated in their capacity by the trainer.

Knowing that cognitive tests performance can be impaired by hypoglycemia. To avoid this bias, patients had their capillary glucose test measured by a glucometer, and the cognitive tests were not administrated if results were below 80 mg/dl. To avoid interference from the environment, the cognitive tests were performed in a silent room and without any unfamiliar person present.

### Statistical analysis

Our outcome variable was cognitive performance and exposure variables were: age, gender, physical activity, tobacco use, alcohol use, any severe hypoglycemia episode in last year, T2DM, PHQ-9 > 9, arterial hypertension, depression/anxiety diagnosis, CVD, use of insulins, use of statins, hypothyroidism, DR, macular edema, diabetic neuropathy, DM kidney disease, BMI ≥ 30 kg/m^2^, eGFR < 60 ml/min/1.73 m^2^, urinary albumin/creatinine ratio > 30 mg/g creatinine, and HBA1c ≥ 7%.

To uniformly analyze the set of different cognitive tests, the results of all tests were transformed into a Z-score, added, and divided by the total number of tests performed to create a continuous variable called Global Cognitive Score [GCS(z)]. Then we categorized it into GCS(z) ≥ or < zero and used it as the outcome variable of interest. The same score was made upon baseline and at the follow-up assessment, using the means and standard deviations (SD) of the initial assessment for each test. Thus, any Z score < 0 at baseline means a worse performance within the group, and in this second assessment indicated a decline in the score compared to the baseline assessment.

Student’s T-Test for dependent samples was used to compare each cognitive test scores and the GCS(z) at baseline and follow-up.

To assure that the sample in follow-up phase was representative of the complete sample at baseline, it was compared demographic, clinical, laboratory characteristics, cognitive tests, and the GCS(z) data between the complete sample at baseline and the sample that participated at follow-up phase, as well as between sample that participated at follow-up and that did not. For that, it was performed the Student’s T-Test for independent samples, the Mann–Whitney test, and Pearson’s chi-square test, when applicable.

The association between GCS(z) and exposure variables was tested using multivariate binary logistic regression at baseline and follow-up. To test the independence of each exposure variable we built the multivariable logistic regression models having other exposure factors as confounders.

These exposure variables were categorized as age $\ge$ 65 years, schooling $\le$ 6 years, PHQ-9 > 9 (yes/no), gender (female/male), physical activity (yes/no), tobacco consumption (current or previous/never), alcohol consumption (current or previous/never), last year severe hypoglycemia episodes (yes/no), DM $\ge$ 10 years (yes/no), arterial hypertension (yes/no), depression/anxiety diagnosis (yes/no), CVD (yes/no), use of insulins (yes/no), use of statins(yes/no), hypothyroidism (yes/no), DR (yes/no), macular edema (yes/no), diabetic neuropathy (yes/no), DM kidney disease (yes/no), BMI ≥ 30 kg/m^2^ (yes/no), eGFR < 60 ml/min/1.73 m^2^ (yes/no) and HBA1c ≥ 7% (yes/no). Only variables with p < 0.25 in the univariate analysis were used in the multivariate models [44]. For all other tests, the significance level used was 5% (SPSS version 22. IBM Corporation, Armonk, NY^®^).

## Results

### Descriptive data

#### Sample characteristics

**Fig. 1:**
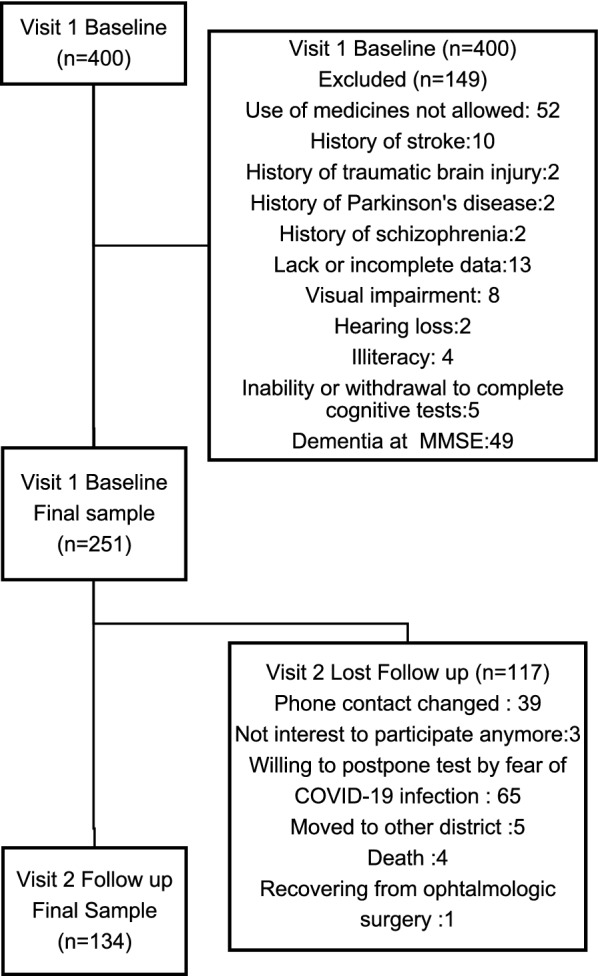
Study flow diagram

Among the 400 patients evaluated, 149 were excluded for several reasons, including 49 (16.3%) that met dementia criteria at MMSE. (Fig. 1).

The final sample consisted of 251 patients, 56.6% female, with a mean age of 61.1 (± 9.8) years, 12.6 (± 8.9) years of DM, and 7.6 (± 4.2) years of school education. Two hundred and six patients (82.4%) were hypertensive and 89% had dyslipidemia. The prevalence of at least one microvascular complication was 54%, with DR being prevalent in 46.5%. Cardiovascular disease was present in 35.2%. In the screening for depression symptoms by the PHQ-9, 37.1% had a score compatible with the risk of major depression and 21.3% had presented at least one episode of severe hypoglycemia in the previous year. DBP value was within the desired range in approximately 36% of the patients and SBP in 65%. (Table 1) (Table 1, Additional file 3).

**Table 1:** Baseline Samples Characteristics

Characteristics	N (134) Mean ± SD/%/Median (IQR)*
Age (years)	61.5 ± 9.9
Education (school years)	7.5 ± 4.3
DM duration (years)	12.5 ± 8.3
Female (%)	52.2
White Race (%)	79.1
Married / Steady Union (%)	61.9
Physically Active (%)	30.6
Smoker/Former smoker (%)	42.5
Alcoholic/Former alcoholic (%)	33.6
Diastolic Blood Pressure (mmHg)	79.6 ± 11.3
Systolic Blood Pressure (mmHg)	129.4 ± 17.8
BMI (Kg/m2)	30.6 ± 4.9
Abdominal circumference (cm)	103.7 ± 12.2
Neck circumference (cm)	39.4 ± 4.2
Arterial hypertension (%)	79.1
Dyslipidemia (%)	92.5
Hypothyroidism (%)	28.4
Hyperthyroidism (%)	3.7
Cardiovascular disease (%)	28.4
Diabetic Retinopathy (%)	44.4
Macular edema (%)	11.1
Diabetic Neuropathy (%)	19.4
DM kidney disease (%)	47.7
Severe hypoglycemia (%)	21.6
Depression/Anxiety	17.9
PHQ-9 score > 9 (%)	39.6
Insulin Use (%)	61.9
Statins Use (%)	79.7
Urea (mg/dl) *	39.0(17.0)
Creatinine (mg/dl)*	0.9(0.4)
eGFR (ml/min/1.73m^2^) *	83.3(31.8)
Blood glucose (mg/dl)*	147.0(71.2)
HbA1c (%)*	7.8(2.3)
ACR (mg/g creatinine) *	17.7(49.6)
TSH (mU/L)*	2.1(1.7)
Free T4 (ng/dl)	1.1 ± 0.3
Triglycerides (mg/dl)*	153.0(119.0)
HDL cholesterol (mg/dl)*	42.0(13.0)
Total cholesterol (mg/dl)*	160.0(57.0)
LDL cholesterol (mg/dl)*	84.0(39.5)
Vitamin B12 (pg/ml)*	340.0(286.0)

*SD* standard deviation, *IQR* interquartile range*, *DM*: diabetes mellitus*,* *BMI*: body mass index, *PHQ*-*9*: patient health questionnaire-9, *eGFR*: estimated glomerular filtration rate, *ACR*: albumin-to-creatinine ratio, TSH: thyroid stimulating hormone, *T4*: thyroxine, *HDL cholesterol*: high density lipoprotein cholesterol, *LDL cholesterol*: low density lipoprotein cholesterol

Among the 134 patients who participated in the follow-up phase, 55% were female, with a mean age of 61.5 (± 9.9) years, 12.5 (± 4.3) years of DM, and 7.5 (± 8.3) years of schooling. One hundred and six patients (79.1%) were hypertensive and 92.5% had dyslipidemia. At least 47.7% had one of the microvascular complications of diabetes, DR was present in 44.4%, and CVD in 28.4%. (Table 1) The average time for performing the reassessment was 18.4 (± 5.0) months after the first phase.

At both times, data from laboratory tests were within normal limits, except for lipid profile, fasting glucose, and HBA1c, with only 30% having HBA1c < 7%. LDL cholesterol levels were within the desired range in 21% of patients and HDL cholesterol in 40%, according to individual stratification of individual risk [28] (Table 1).

There was no significant difference in demographic, clinical, laboratory aspects, and cognitive tests at baseline between the complete sample, the sample that participated in the follow-up phase, and the sample that did not (Tables 1 and 2, Additional file 3).

**Table 2:** Baseline cognitive tests and global cognitive score (z)

Characteristics	N = 134 mean ± SD/%
MMSE (score)	27.2 ± 2.0
Verbal fluency (score)	16.7 ± 5.0
TMTA (seconds)	55.3 ± 26.4
TMTB (seconds)	162.6 ± 109.6
Immediate Memory (score)	16.5 ± 4.1
Recall Memory (score)	5.1 ± 1.9
Recognition Memory (score)	8.4 ± 1.7
GCS(z) (score)	0.092 ± 0.631
GCS(z) < 0 (%)	41.8

Student-T test for paired samples

*SD* standard deviation, *MMSE*: Mini-Mental State Exam, *TMT* *A* and *B* trial making test A and B, *GCS(z)* global cognitive score (z)

### Cognitive tests and GCS(z) results

The cognitive tests and the GCS(z) results at baseline are resumed in Table 2.

### Main results

Although we did not find a significant difference between the means of the cognitive tests scores and GCS(z) at the two evaluation moments (Table 3), eleven (14.1%) patients with GCS ≥ 0 at baseline had GCS < zero at the 18 months follow-up period, with a mean z score decrease of -0.439 ± 0.255.

**Table 3:** Comparison of cognitive tests and global cognitive scores at baseline and follow-up

Cognitive Tests	Baseline (n = 134) Mean ± SD	Follow-up (n = 134) Mean ± SD	P
MMSE (score)	27.2 ± 2.0	27.1 ± 2.1	0.57
Verbal Fluency (score)	16.7 ± 5.0	16.4 ± 5.0	0.28
TMTA (seconds)	55.2 ± 26.5	55.8 ± 30.4	0.71
TMTB (seconds)	154.9 ± 109.40	152.5 ± 121.6	0.77
Immediate Memory (score)	16.5 ± 4.2	16.5 ± 4.2	0.95
Recall Memory (score)	5.1 ± 1.9	5.0 ± 2.0	0.68
Recognition Memory (score)	8.4 ± 1.7	8.4 ± 1.7	0.96
GCS(z)	0.092 ± 0.631	0.068 ± 0.699	0.51

Student-T test for paired samples

*SD* Standard deviation, *MMSE* Mini-Mental State Exam, *TMT* *A* and *B* Trail making test A and B, *GCS(z)* Global cognitive score (z)

**Fig. 2:**
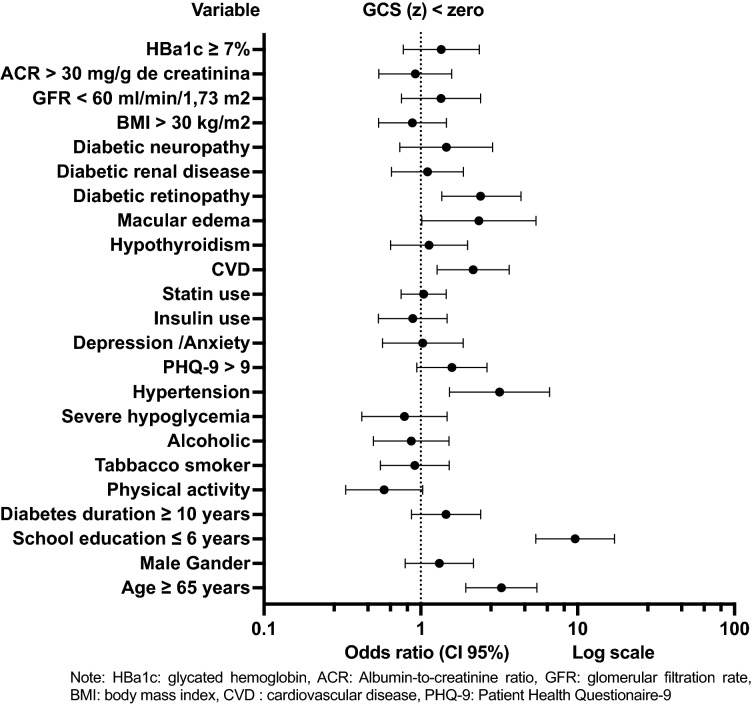
Baseline exposure factors for Global Cognitive Score < 0 Univariate Binary Logistic Regression

At baseline, multivariate binary logistic regression analysis was built having the exposure variables extracted from the univariate binary logistic regression analysis. These variables were age ≥ 65 years, schooling $\le$ 6 years, score on the PHQ-9 questionnaire > 9, DM duration ≥ 10 years, physical activity, arterial hypertension, CVD, DR, and macular edema (Fig. 2) (Additional file 4).

**Fig. 3:**
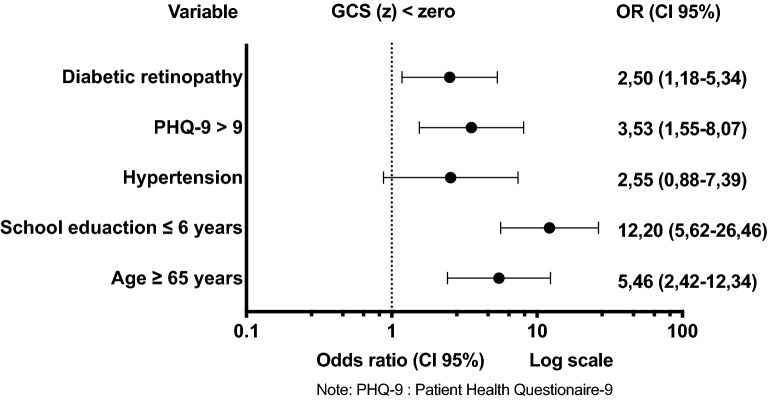
Baseline exposure factors for Global Cognitive Score < 0 Multivariate Logistic Regression

Multivariate logistic regression model at baseline resulted in five exposure variables associated with GCS(z) < 0: age ≥ 65 years, ≤ 6 school education years, the presence of arterial hypertension, score on the PHQ-9 questionnaire > 9, and DR (Fig. 3) (Additional file 4) even after adjustment for DM duration ≥ 10 years, physical activity and CVD.

**Fig. 4:**
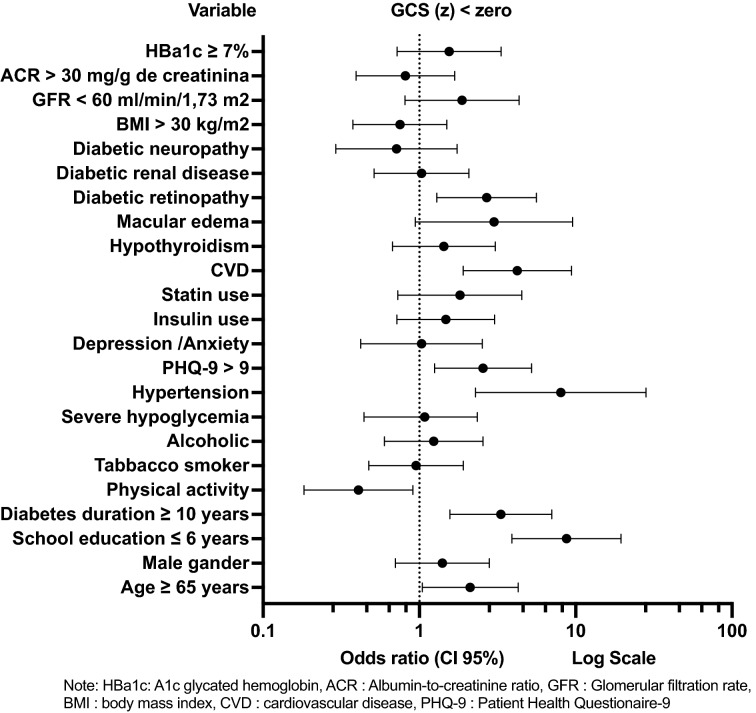
Follow-up exposure factors for Global Cognitive Score < 0 Univariate Logistic Regression

In follow-up, we also built a multivariate binary logistic regression analysis having the exposure variables extracted by univariate binary logistic regression analysis. These variables were, age ≥ 65 years, schooling $\le$ 6 years, score on the PHQ-9 questionnaire > 9, physical activity, DM duration $\ge$ 10 years, arterial hypertension, CVD, DR, macular edema, use of statins, and GFR < 60 ml/minute/1.73 m^2^. (Fig. 4) (Additional file 5).

**Fig. 5:**
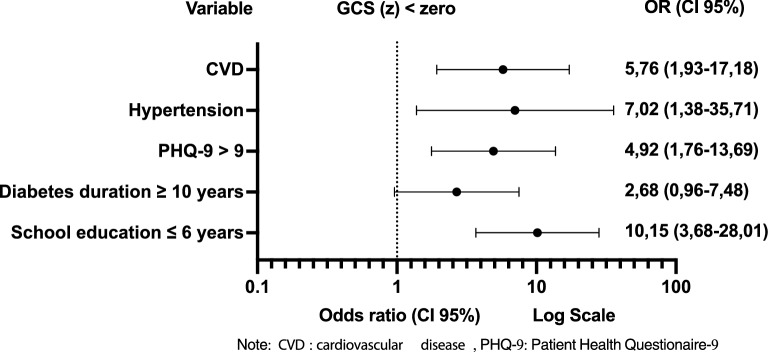
Follow-up exposure factors for Global Cognitive Score < 0 Multivariate Logistic Regression

The multivariate logistic regression model resulted in five explanatory variables associated with GCS(z) < 0 at follow-up: school education years $\le$ 6 years, DM duration $\ge$ 10 years, score on the PHQ-9 questionnaire > 9, arterial hypertension, and CVD, even after adjustment for age $\ge$ 65 years, physical activity, DR, macular edema, use of statins and GFR < 60 ml/minute/1.73 m^2^. (Fig. 5) (Additional file 5).

## Discussion

Modifiable risk factors that were significantly associated with lower cognitive performance at baseline and in the follow-up after adjustments, were prolonged DM duration, depression symptoms, arterial hypertension, CVD, and DR.

Other cross-section studies also demonstrated that arterial hypertension is associated with cognitive decline and dementia [45, 46]. Epidemiological and longitudinal studies found an association between hypertension in middle age with incident dementia and cognitive decline later in life in the general population, but this association in less robust if hypertension initiates in later life [47–53].

Hypertension is well established as a risk factor for cardiovascular and cerebrovascular disease. Especially in midlife, it has been identified as a risk factor for cerebral atrophy, white matter microstructural damage, stroke, and cerebral small vessel disease. There is also evidence of an association between vascular dementia and white matter hyperintensities progression, cerebral microbleeds, and lacunar infarcts [51, 53]. Evidence suggests that hypertension contributes to the development and progression of such neurological changes by promoting vessel wall remodeling and endothelial dysfunction [55–57]. AD is also associated with hypertension, demonstrated by the accentuation of reduced brain volume and increase of β-amyloid plaques and neurofibrillary tangles in hypertensive patients compared with normotensives [58, 59].

So, it seems clear that hypertension can affect brain structure and function, but these associations are influenced by many concurrent factors like age, duration of hypertension, intensity of blood pressure control, and specific mediations. A controversial issue is whether the intensity and type of medication used for hypertension treatment could interfere with cognitive outcomes still exist. A recent meta-analysis concluded that adequate antihypertensive treatment reduces the incidence of dementia and MCD in the long term [60]. The Sprint Mind study showed a reduction in the risk of MCD in patients adequately treated for hypertension [61]. Although some studies suggest better cognitive performance with specific antihypertensive classes, a recent review failed to show a benefit of one antihypertensive class over another [62]. Therefore, efforts for early arterial hypertension diagnosis and adequate treatment according to international guidelines in middle age seem a reasonable protective action in high-risk patients.

Diabetes and depression are frequently concomitant. Individuals with type 2 diabetes have a doubled risk for depression as compared with individuals without diabetes, otherwise, individuals with depression have a 1.5 higher risk of diabetes [63]. This correlation is complex and the pathophysiologic pathway behind this association is not completely understood, but hyperglycemia, microvascular dysfunction, dysregulation of the hypothalamic-pituitary axis, and low-grade inflammation are abnormalities that can explain the temporal association between depression and type 2 diabetes [64, 65]. Depression is closely associated with cognitive dysfunction and dementia, but it is still unclear to what extent it is a risk factor or whether it is a prodromal symptom of dementia. Some studies associate the presence of depression as a risk factor when it starts at young or middle age, and as a prodromal symptom of dementia when it starts in later life [2, 66–68]. Therefore, the presence of depression symptoms at a later age should be seen as a warning sign for the later development of dementia. In this study, there was a significant association between the score of depression symptoms (PHQ-9 > 9) and worse cognitive performance at baseline and in the follow-up period. It is not possible to know whether it is a symptom or a risk factor, reinforcing the importance of diagnosing and treating depression, as well as monitoring cognitive decline in these patients with depression symptoms.

CVD was present in 35% of the patients in this study and was one of the risk factors associated with worse cognitive performance in the follow-up. The Brazilian cross-section brain bank study that explored neuropathologic lesions and cognitive status, found that neuropathological diagnosis irrespective of cognitive status was found in 44% of patients. Among them, 20% had mixed neuropathological findings. Vascular dementia (VD) was present in 35% and Alzheimer’s disease (AD) alone or associated with another neuropathological diagnosis in 50%. These findings show that vascular disease is frequent in this population, showing the importance of cardiovascular risk factors and cardiovascular disease [69].

In a large Taiwanese cohort, the development of any vascular event, especially in the first years after the diagnosis of T2DM, showed a risk for dementia up to twice as high [70]. Another longitudinal study explored the association between cardiovascular health score at age 50 and incidence of dementia and found that adherence to ideal cardiovascular vascular health recommendations (smoking, diet, physical activity, body mass index, fasting glucose, blood cholesterol, blood pressure) in midlife was associated with a lower risk of dementia later in life [71]. Exalto L.G. et al. created a 10-year dementia risk score and found that: microvascular disease, diabetic foot, cerebrovascular disease, cardiovascular disease, acute metabolic events, depression, age, and education most strongly predicted dementia [72].

There is some evidence that a decrease of cerebral blood flow (CBF) causes a series of changes in the neurovascular unit (NVU), such as impaired neuronal function, abnormal activation of glial cells, and changes in vascular permeability, all of which collectively play a role in the pathogenesis of VD [73, 74].

In 2020, The Lancet Commission on dementia prevention, intervention, and care, supported a growing body of evidence for eleven potentially modifiable risk factors for dementia: less education, hypertension, hearing impairment, smoking, obesity, depression, physical inactivity, diabetes, low social contact, excessive alcohol consumption, traumatic brain injury, and air pollution. According to this commission, the CVD risk factors: hypertension, smoking, obesity, sedentarism and diabetes should be prevented and controlled to prevent dementia [75]. However, adequate control of these cardiovascular factors is far from what National and International Guidelines recommend, despite being widely publicized and disseminated by Medical Societies and Health Management Bodies worldwide [22, 76–79]. This study also identified a high percentage of patients with uncontrolled cardiovascular risk factors, which highlights the need to identify barriers from the point of view of health professionals, managers, and patients preventing achieving these goals.

DR was identified as the other risk factor for worse cognitive performance. Recently a systematic review and meta-analysis of twenty-two studies, including cross-sectional and cohort studies showed a similar association between DR and cognitive impairment. In this study, the presence of DR reflected a higher cognitive dysfunction with OR = 2.45 (95% CI 1.76–3.41) and HR = 1.34 (95% CI 1.10–1.62). The pooled OR was 2.38 and 3.11 for Asia and Oceania respectively, and there was no association in North America and with T1DM. There was no study from South and Central America in this review. They found as well that DR severity showed a positive correlation with cognitive impairment [80]. One other review and meta-analysis evaluated the association between DR and cerebral small vessel disease with any type of cognitive dysfunction and found an association between DR and structural abnormalities in the brain with impaired cognitive function [81].

Retina and brain have similar pathological and aging mechanisms and, therefore, can be an easily accessible source of information for cerebral neurodegenerative processes. Communication between the brain and the retina occurs through retinal ganglion cells, connecting with the cortex through the optic nerve. Like the blood–brain barrier, the blood-retinal barrier plays a role in regulating the supply of oxygen and glucose and protecting the retinal microenvironment against exposure to molecules, in this case, especially to inflammatory cytokines commonly circulating in patients with DM and its comorbidities [82, 83]. The production or activation of inflammatory cytokines at the brain level can lead to insulin action resistance in the brain, resulting in deterioration of brain processes such as neuron survival, dendritic plasticity, synaptic function, learning, and memory [84, 85].

Optical coherence tomography and corneal microscopy are non-invasive, rapid assessment tests that can produce retinal and corneal images and detect findings of neurodegeneration. The retinal nerve fiber layer is the innermost part of the retina and is formed by retinal ganglion cells and alterations in this region are associated with neurodegeneration [86–88]. Retinal microcirculation can also be non-invasively visualized using retinal arteriography [89]. Studies have observed an association between corneal neurodegeneration findings with cognitive alterations and imaging findings related to MCD and dementia [90–92]. Both microcirculatory and neurodegenerative changes in the retina and cornea are associated with cognitive changes and dementia, but the better marker and the mechanisms involved have not been fully elucidated [83].

One recent review studied the risk factors for progression from MCD to dementia in a T2DM population and found an association between longer DM duration and DR with increased risk for cognitive decline progression [93]. The mechanisms by which T2DM acts as an accelerating factor in the dementia process are not fully known. Several pathogenic mechanisms have been postulated, including hyperglycemia and its enzymatic glycation end products, insulin resistance, hypoglycemia, micro and macrovascular disease, inflammation, oxidative stress, in addition to alterations in the metabolism of amyloid peptides, amylin, and tau protein. It is most likely that these mechanisms are concurrent and occur in different proportions in different patients, but insulin resistance and inflammation may be the connection of several of these mechanisms [47–49].

DM courses with alterations in the blood–brain barrier and microvascular dysfunctions, making the brain susceptible to various aggressions and compromising the supply of nutrients [50]. An in vivo study with animal models of T1DM and T2DM showed that the breakdown of this barrier was associated with increased expression of inflammatory genes at the brain level [51]. Patients with insulin resistance, prediabetes, and diabetes have activation of tumor necrosis factor-α resulting in inhibition of phosphorylation of the tyrosine kinase enzyme in insulin receptors in the periphery, and this is one of the main mechanisms of peripheral insulin resistance [52]. Similarly, the production or activation of inflammatory cytokines at the brain level can lead to insulin action resistance in the brain, resulting in deterioration of brain processes such as neuron survival, dendritic plasticity, and synaptic function, learning, and memory [51, 53, 54].

Some limitations can be pointed out in this study. One of them is the lack of imaging tests to correlate with the clinical results. One other limitation was the number of patients lost at follow-up phase. The follow-up sample could lead to selection bias if their characteristics differed from the baseline sample. However, we have tested potential differences among those who participated and those who did not participate in the follow-up, and no difference was found, as well as checked critical reasons for loss of follow-up and did not find any. We also believe that despite our efforts in using validated instruments for cognitive assessment applied by trained specialists, recall bias can be present.

The strength of this study is that there are few studies in Brazil evaluating cognition, cognitive decline, dementia, and related risk factors in the general population and even less in the T2DM population [40, 41, 94]. This is possibly the first national longitudinal study to clinically assess cognitive dysfunctions in the T2DM from a public health system and its risk factors. Even with the limitations, we reproduced data from a real-life scenario in public health in a middle-low-income country setting that could be generalized for similar health services around the world, since the last IDF Atlas indicates that 4 in 5 people with diabetes are at low and middle-low-income countries [6].

Our findings confirm the importance of screening for cognitive dysfunction and dementia in T2DM population. Brazilian and the American Diabetes Society/ Endocrine Society guidelines recommend the individualization of goals and choices for DM treatment in the elderly based on their self-care capacity and cognitive status, improving the quality of care for these patients [20, 95, 96].

## Conclusions

Based on our results, cognitive evaluation and follow-up should be incorporated into the routine of T2DM patients, especially for those with advanced age, low education level, prolonged DM duration, arterial hypertension, depression symptoms, CVD, and DR.

Even though guidelines already suggest the need for screening for cognitive dysfunctions in the T2DM population over 60 years of age, this does not happen in clinical practice due to the difficulties in carrying out time-consuming tests that require training to be performed. However, identifying and following those at higher risk for cognitive dysfunction and decline could help clinicians to improve the quality of care for these patients and concentrate efforts to retard cognitive decline.

## Supplementary Information

**Additional file 1.** Cognitive tests

**Additional file 2.** PHQ-9

**Additional file 3:** **Table 1.** Baseline Samples Characteristics: Comparison between sample that participated follow-up and sample that did not participated follow-up. **Table 2.** Baseline Samples Characteristics: Comparison between complete sample and sample that participated follow-up

**Additional file 4:** **Table 1.** Baseline Univariate Binary Logistic Regression Model - GCS < 0 and exposure variables*. **Table 2.** Baseline Multivariate Binary Logistic Regression Model - GCS < 0 and exposure variables*. **Table 3.** Follow-up Univariate Binary Logistic Regression Model - GCS < 0 and exposure variables*. **Table 4.** Follow-up Multivariate Binary Logistic Regression Model - GCS < 0 and exposure variables*

**Additional file 5.** Research ethics committee approval letter

## Data Availability

Most data generated or analyzed during this study is included within the article and its supplementary information file. Any additional data is available from the corresponding author on reasonable request.

## Abbreviations

HBA1c: Glycated hemoglobin
DM: Diabetes mellitus
T2DM: Type 2 Diabetes mellitus
T1DM: Type 1 Diabetes mellitus
MCD: Minimal cognitive decline or dysfunction
CVD: Cardiovascular disease
GCS(z): Global cognitive score
AH: Arterial hypertension
HDL: High-density lipoprotein
BMI: Body mass index
LDL: Low-density lipoprotein
MMSE: Mini-Mental State Examination
DBP: Diastolic blood pressure
SBP: Systolic blood pressure
PHQ-9: Patient Health Questionaire-9
ACR: Albumin-to-creatinine ratio
DR: Diabetic retinopathy
eGFR: Estimated Glomerular filtration rate
VD: Vascular dementia
AD: Alzheimer disease
CBF: Cerebral blood flow
NVU: Neurovascular unit
