# Supplementary material for: Risk factors for cognitive decline in type 2 diabetes mellitus patients in Brazil: a prospective observational study
Source: Diabetol Metab Syndr. 2022 Jul 27;14:105. doi: 10.1186/s13098-022-00872-3 (PMC9327152; doi:10.1186/s13098-022-00872-3)

**TRAIL MAKING TEST A**


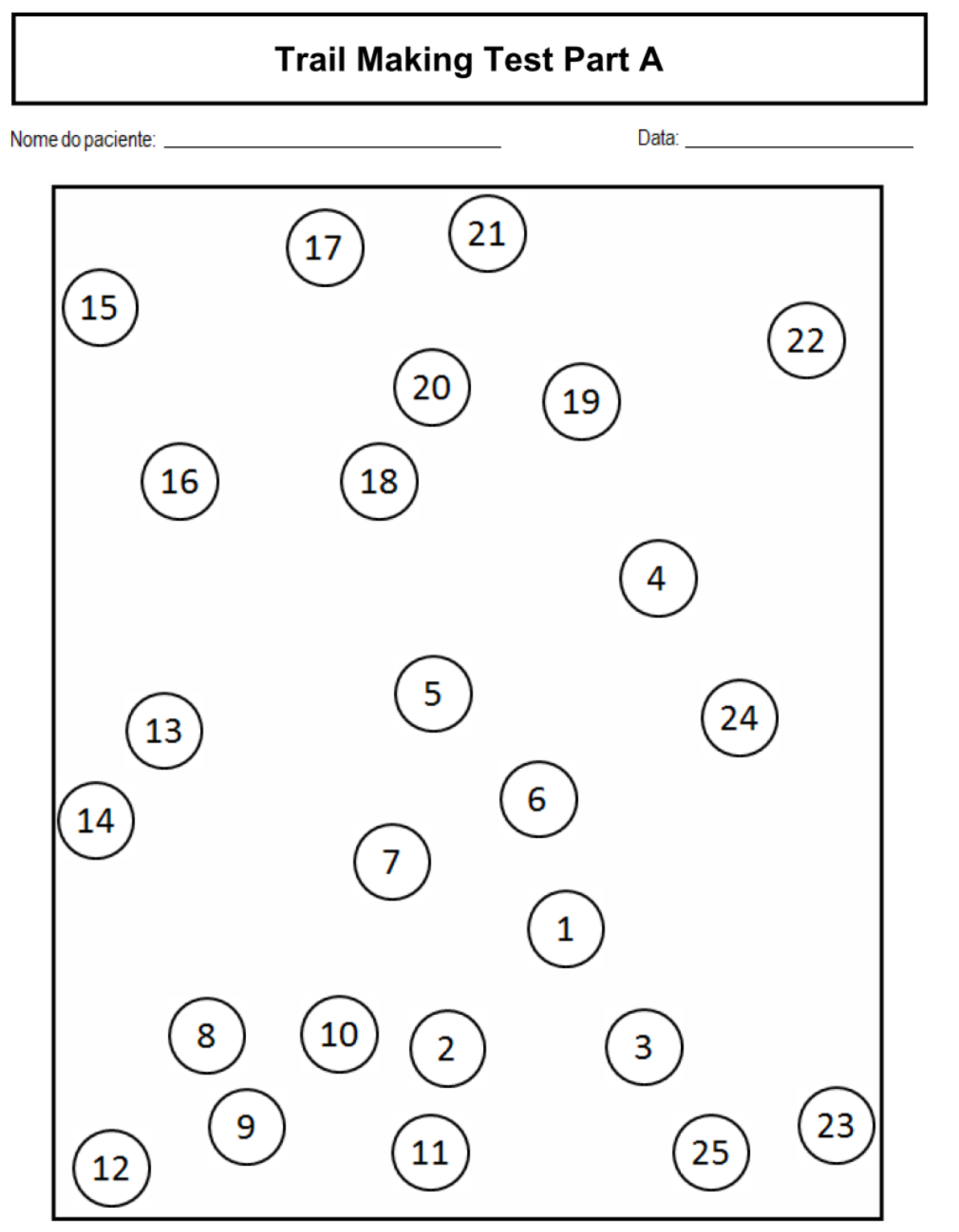


**TRAIL MAKING TEST B**


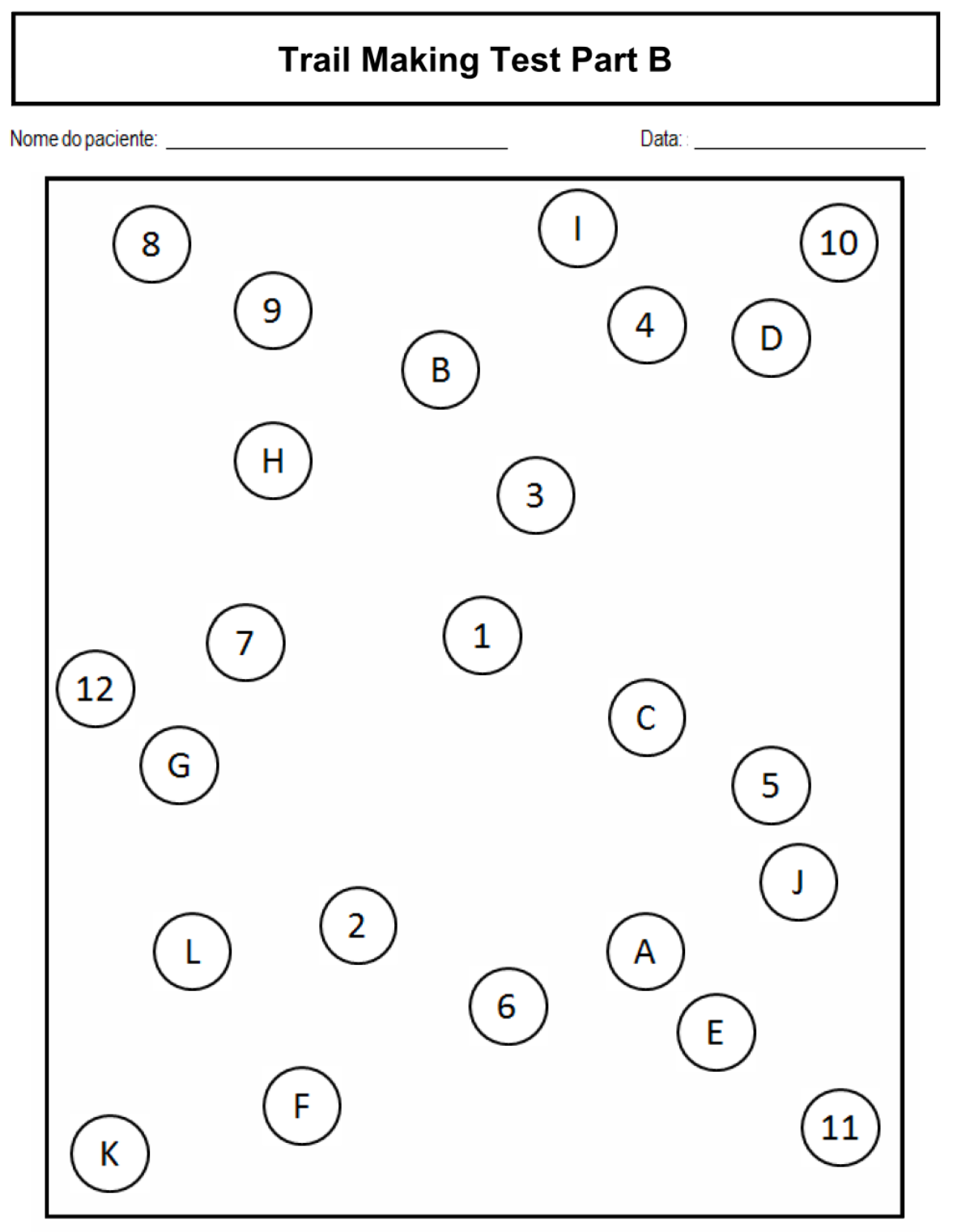


**SEMANTIC VERBAL FUENCY TEST**


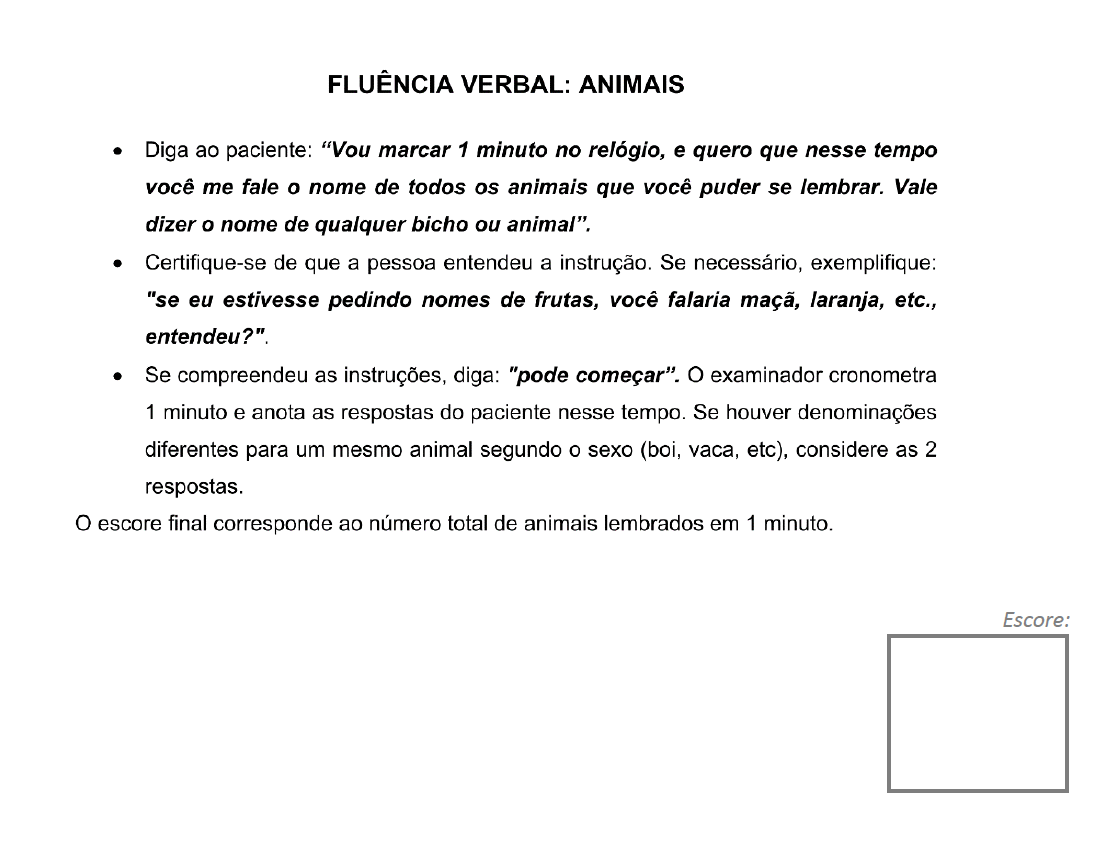


**WORD TEST LIST**


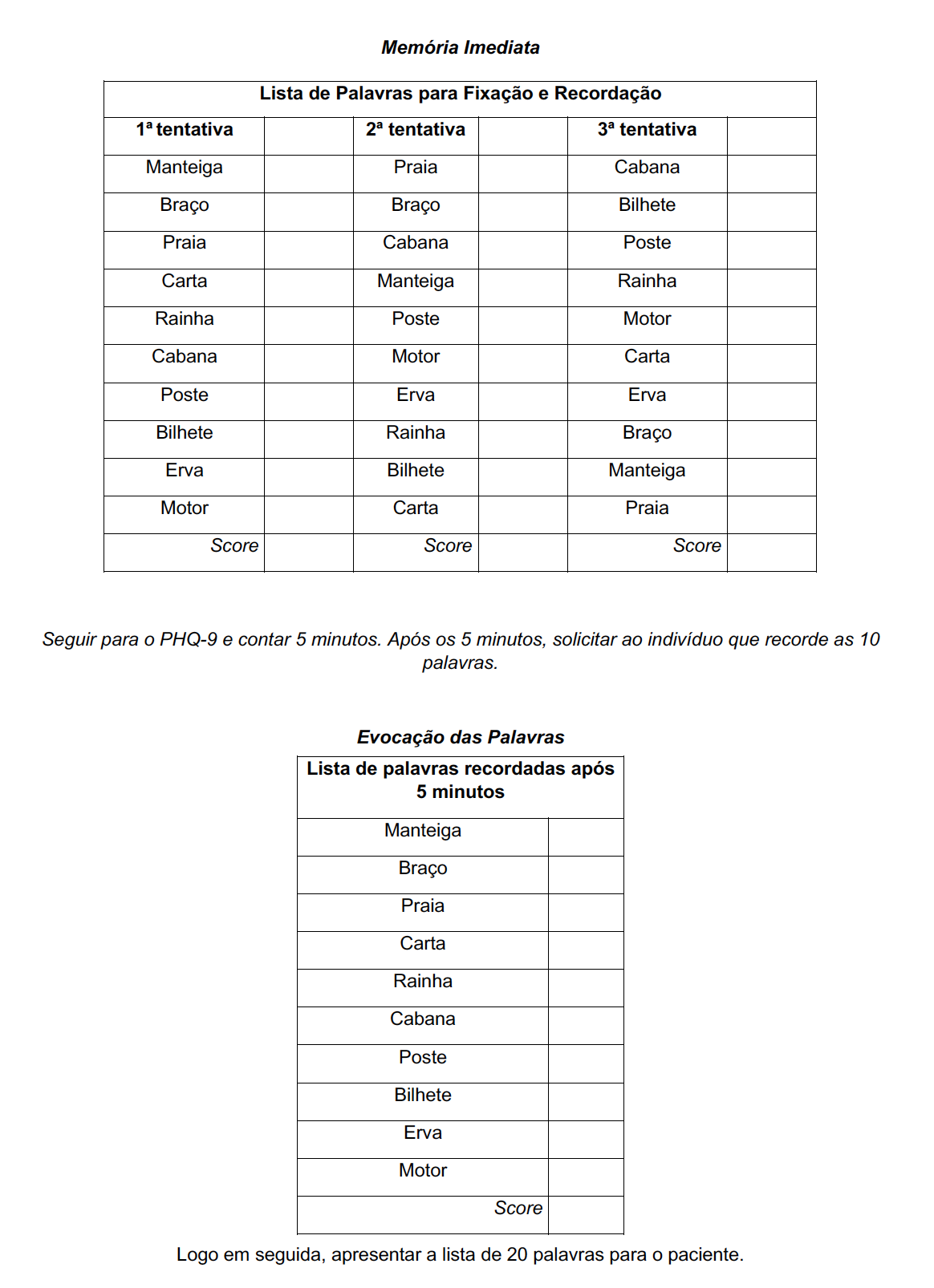


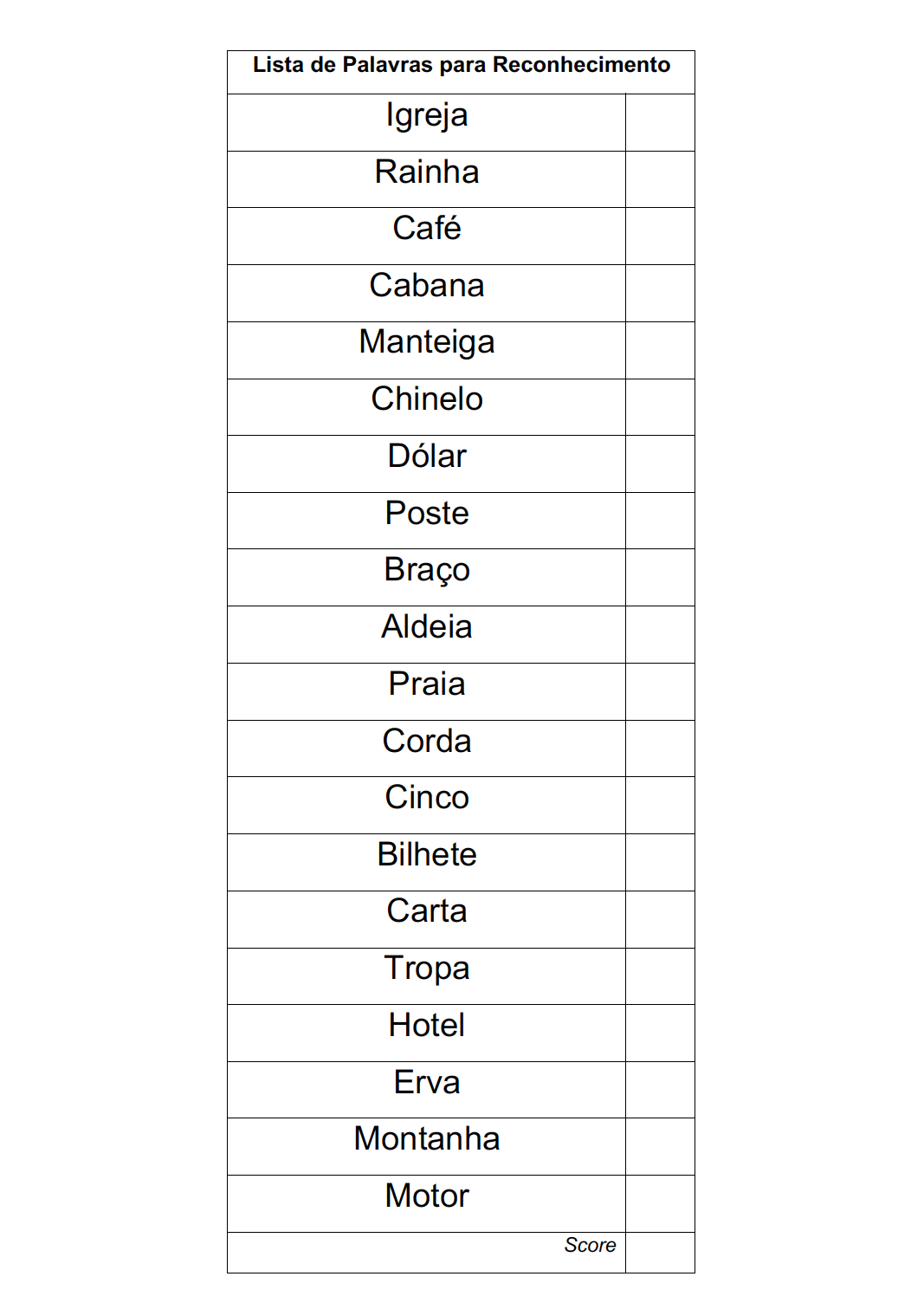


**MINI MENTAL STATE EXAM**


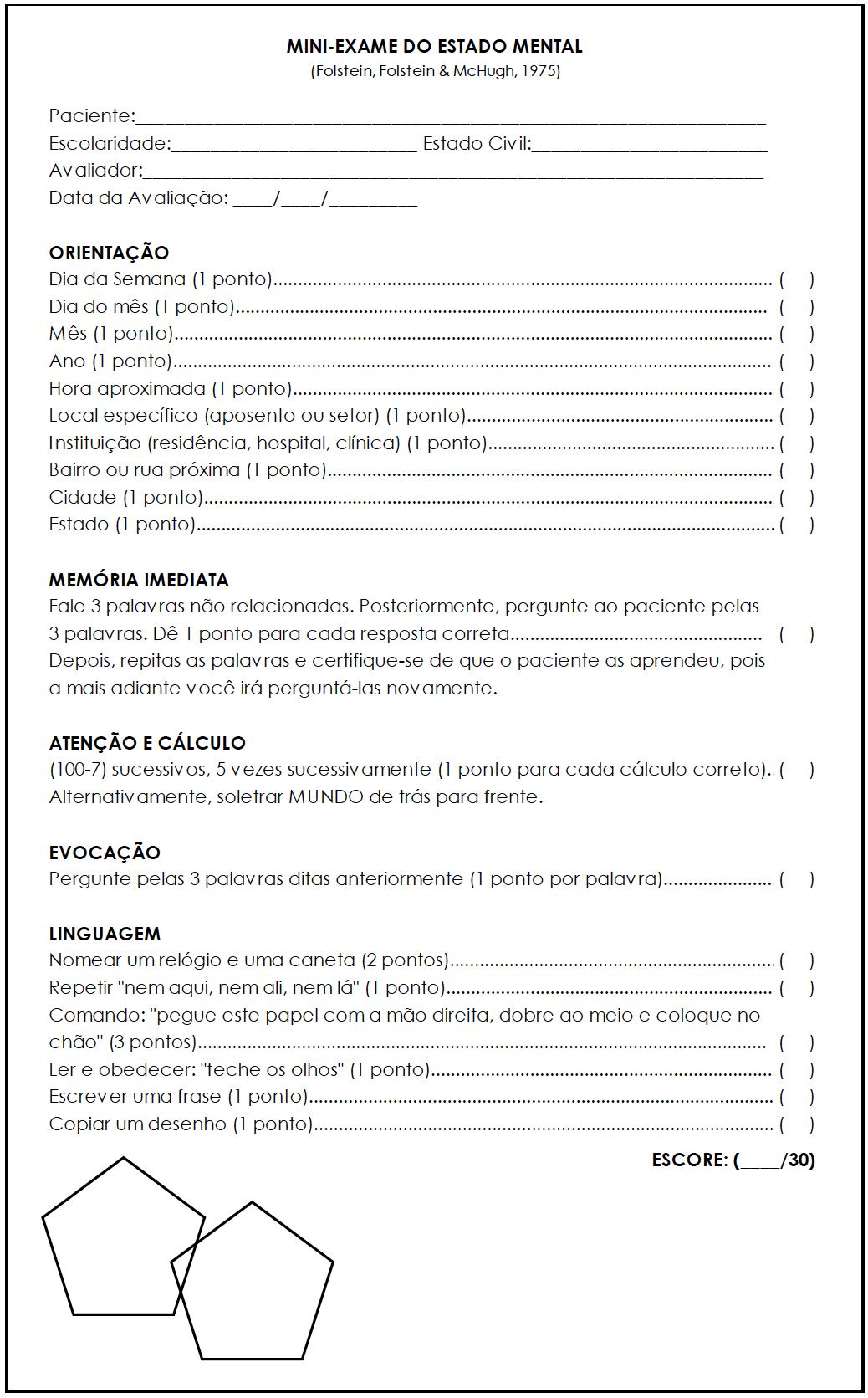

Supplement: Supplementary file 1 — Additional file 1. Cognitive tests [file 13098_2022_872_MOESM1_ESM.docx]
