# Supplementary figures and images for: Risk factors for cognitive decline in type 2 diabetes mellitus patients in Brazil: a prospective observational study
Source: Diabetol Metab Syndr. 2022 Jul 27;14:105. doi: 10.1186/s13098-022-00872-3 (PMC9327152; doi:10.1186/s13098-022-00872-3)

**PATIENT HEALTH QUESTIONNARIE-9 (PHQ-9)**


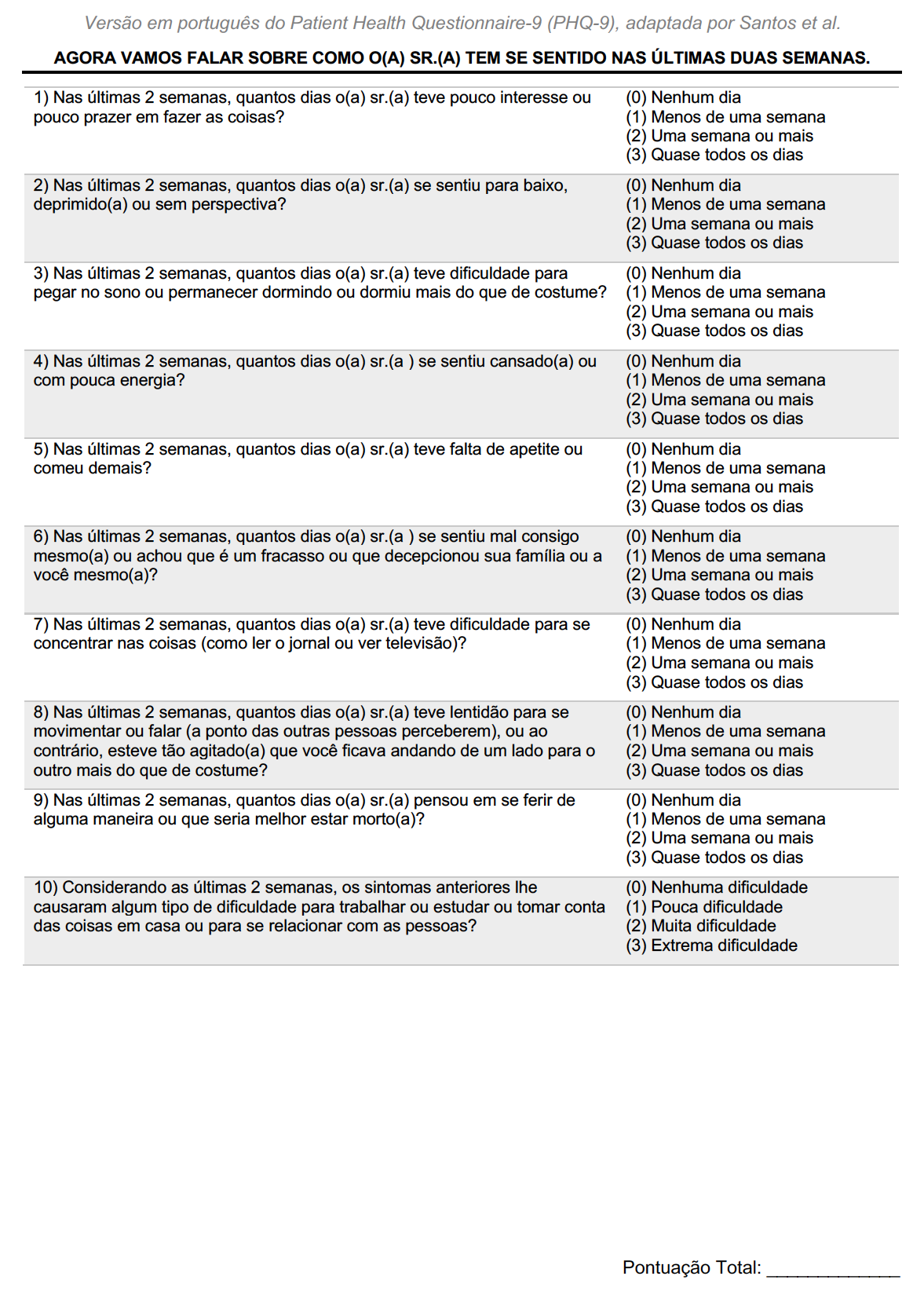


A

Supplement: Supplementary file 2 — Additional file 2. PHQ-9 [file 13098_2022_872_MOESM2_ESM.docx]

**Research Ethics Committee of the Catholic University of Paraná**

**Approval Letter**
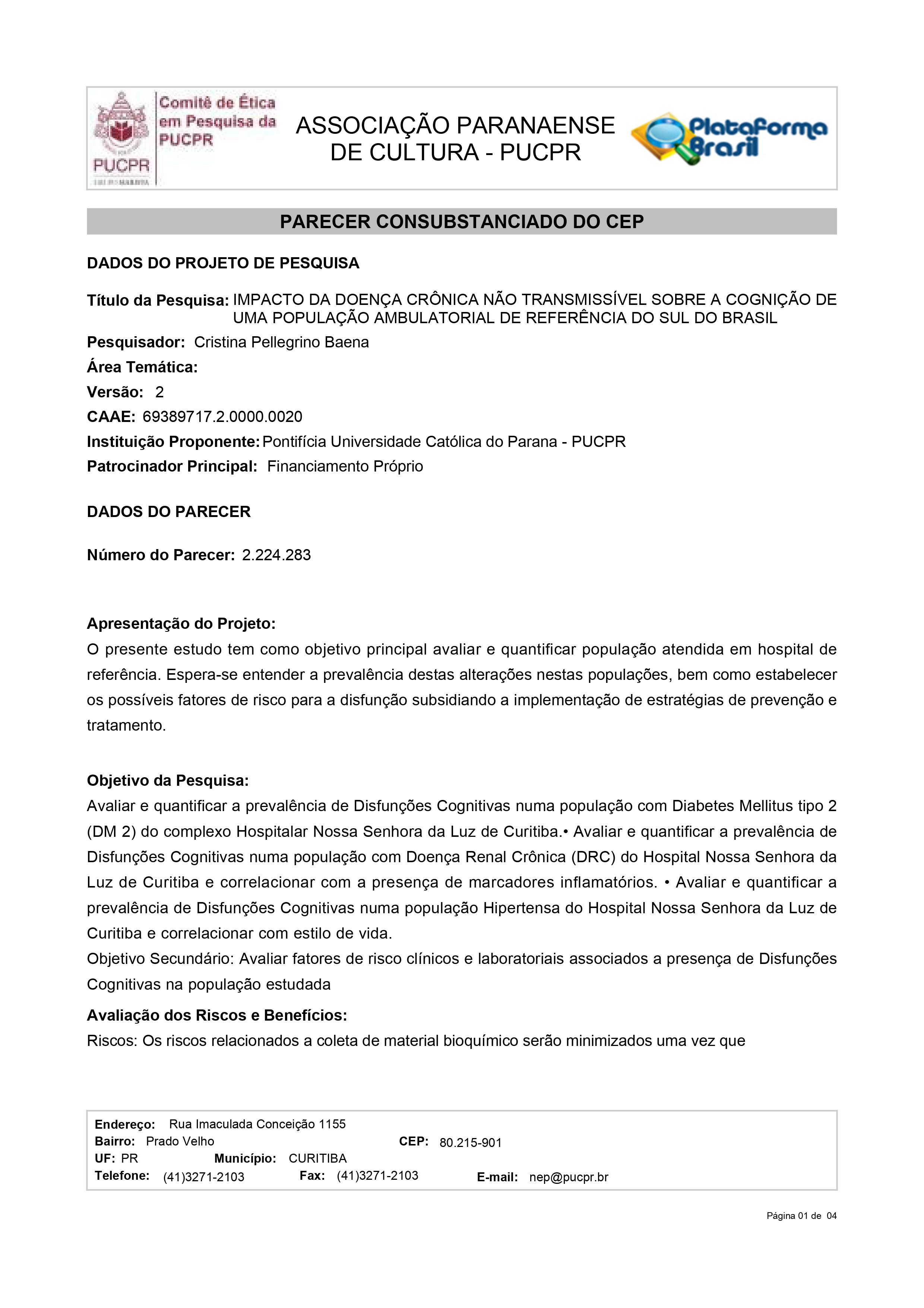


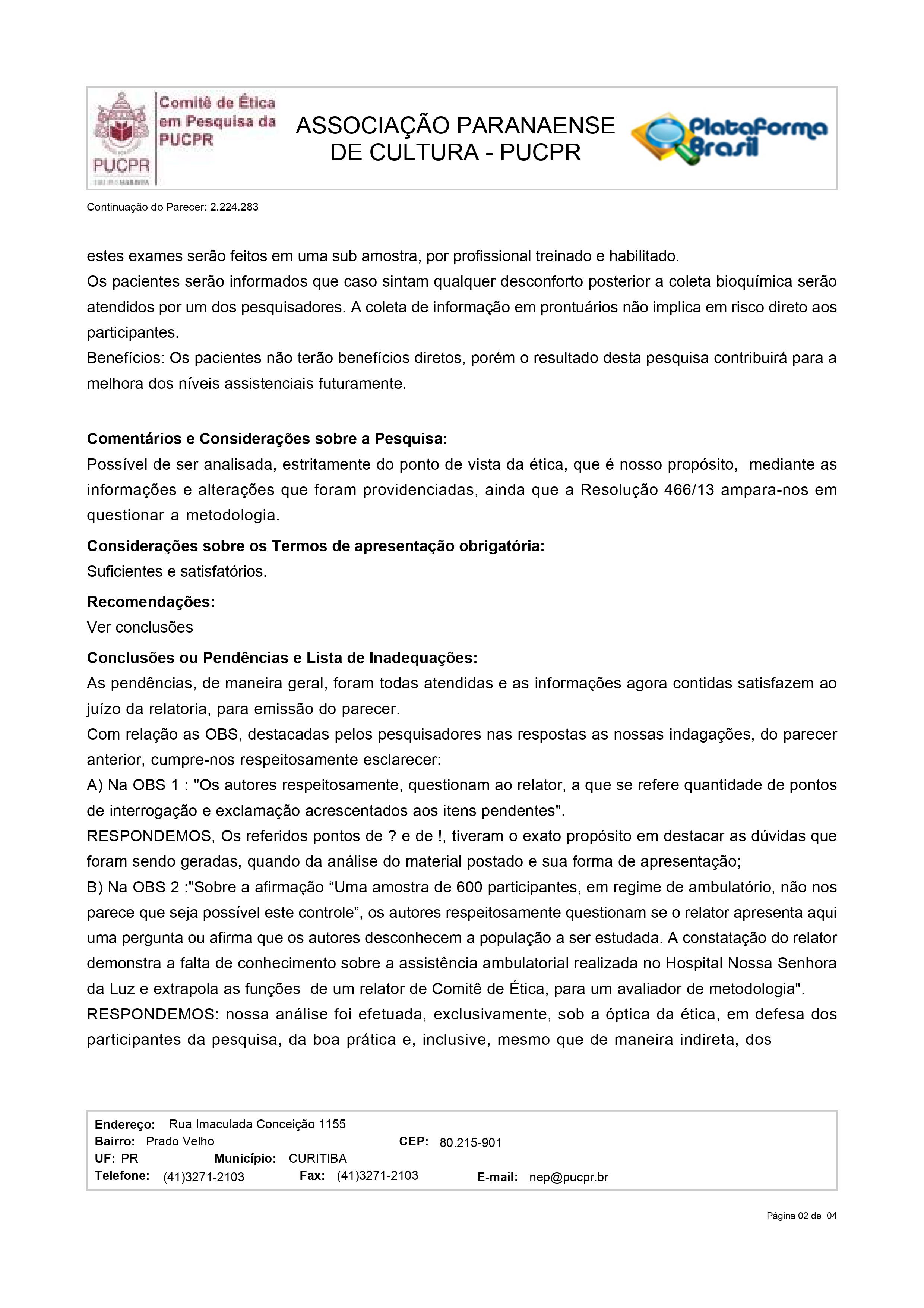

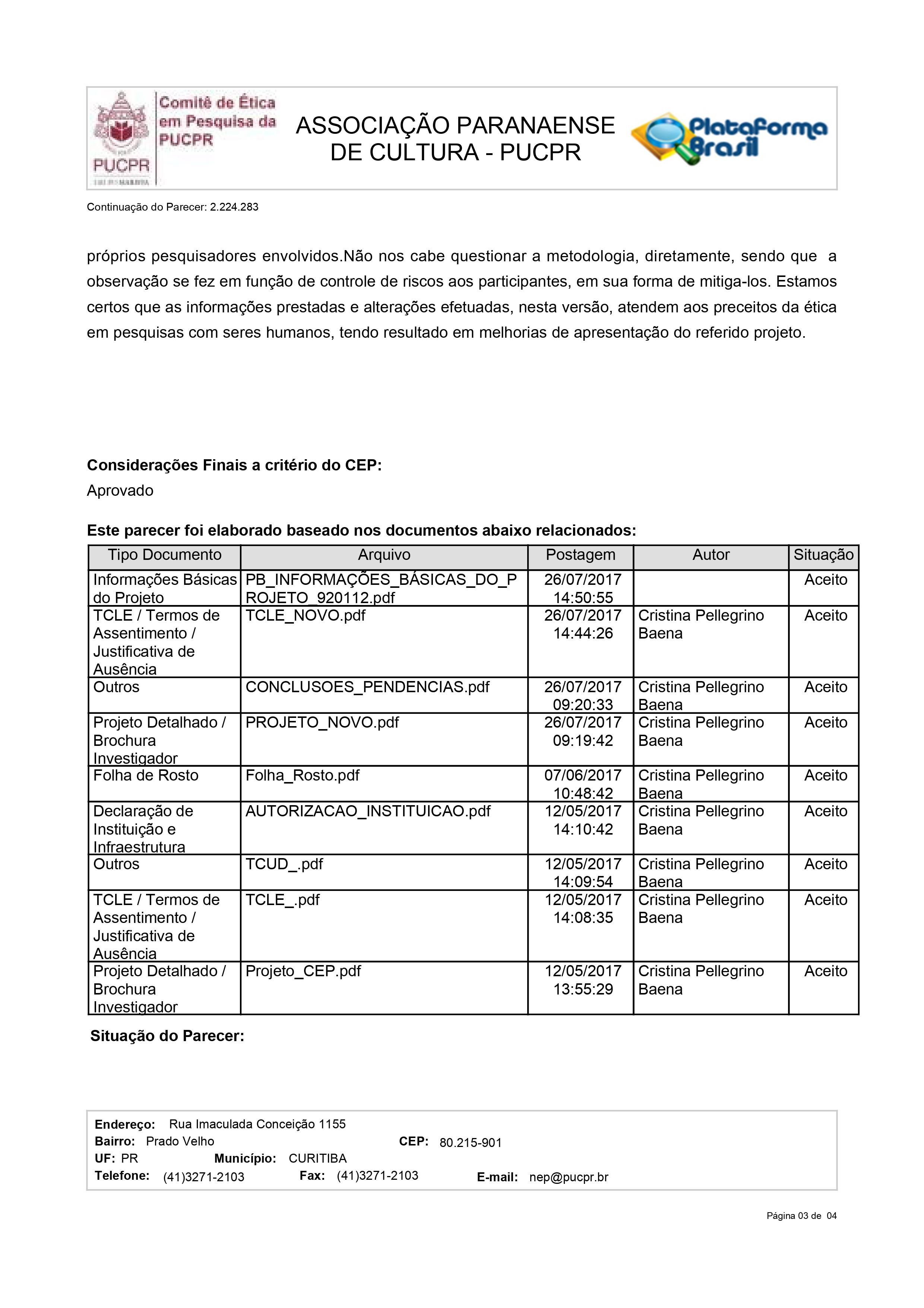

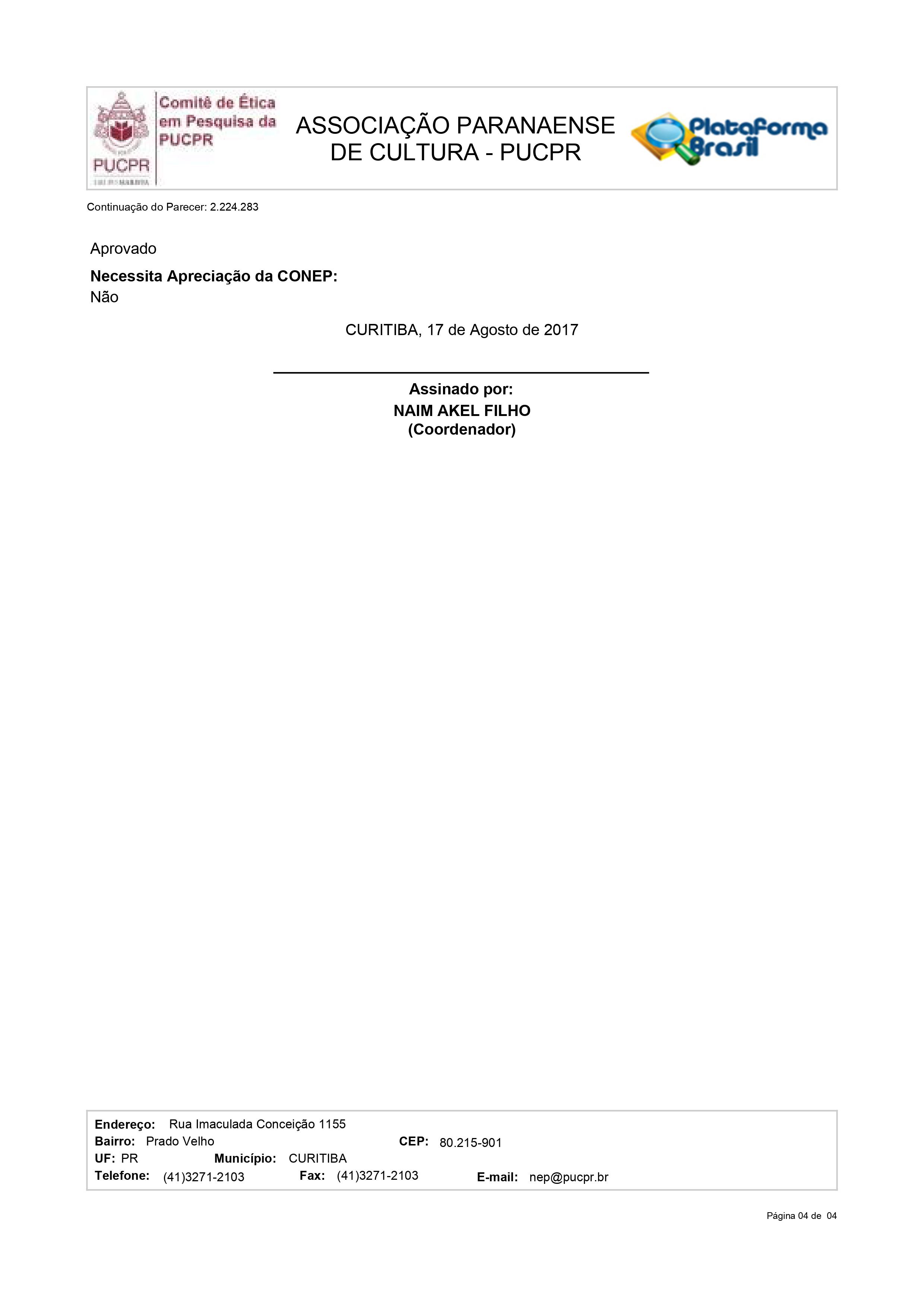

Supplement: Supplementary file 5 — Additional file 5. Research ethics committee approval letter [file 13098_2022_872_MOESM5_ESM.docx]
