# Supplementary material for: Risk factors for cognitive decline in type 2 diabetes mellitus patients in Brazil: a prospective observational study
Source: Diabetol Metab Syndr. 2022 Jul 27;14:105. doi: 10.1186/s13098-022-00872-3 (PMC9327152; doi:10.1186/s13098-022-00872-3)
Supplement: Supplementary file 4 — Additional file 4: Table 1. Baseline Univariate Binary Logistic Regression Model - GCS < 0 and exposure variables*. Table 2. Baseline Multivariate Binary Logistic Regression Model - GCS < 0 and exposure variables*. Table 3. Follow-up Univariate Binary Logistic Regression Model - GCS < 0 and exposure variables*. Table 4. Follow-up Multivariate Binary Logistic Regression Model - GCS < 0 and exposure variables* [file 13098_2022_872_MOESM4_ESM.docx]

**Table 1: Baseline Univariate Binary Logistic Regression Model - GCS < 0 and explanatory variables***

| Variable | Classification | n | GCS < 0  n (%) | P* | Odds Ratio (CI 95%) |
| --- | --- | --- | --- | --- | --- |
| **Age (years)** | $\geq$ 65 | 106 | 67 (57.3) | 0.000 | 3.264 (1.936 - 5.505) |
|  | $<$ 65 | 145 | 50 (42.7) |  |  |
| **School education (years)** | $\leq$6 | 118 | 87 (74.4) | 0.000 | 9.635(5.40917.165) |
|  | $>$ 6 | 133 | 30 (25.6) |  |  |
| **Physical activity** | Yes | 70 | 26 (22.2) | 0.063 | 0.584 (0.332– 1.029) |
|  | No | 181 | 44 (32.8) |  |  |
| **Tabbacco smoker** | Yes | 115 | 51 (45.9) | 0.735 | 0.916 (0.553 – 1.518) |
|  | No | 129 | 60 (54.1) |  |  |
| **Alcoholic** | Yes | 72 | 31 (27.9) | 0.621 | 0.870 (0.499 – 1.514) |
|  | No | 172 | 80 (72.1) |  |  |
| **Diabetes duration (years)** | Yes | 149 | 75 (64.1) | 0.154 | 1.448 (0.871 – 2.408) |
|  | No | 102 | 42 (35.9) |  |  |
| **Severe hypoglycemia** | Yes | 51 | 21 (19.1) | 0.453 | 0.787 (0.420 – 1.472) |
|  | No | 189 | 89 (80.9) |  |  |
| \| **Hypertension** \| \| --- \| | Yes | 206 | 106(90.6) | 0.002 | 3.180 (1.525 – 6.632) |
|  | No | 44 | 11 (9.4) |  |  |
| **PHQ-9 > 9** | Yes | 93 | 50 (42.7) | 0.082 | 1.579 (0.943 – 2.644) |
|  | No | 158 | 67 (57.3) |  |  |
| \| **Depression /Anxiety** \| \| --- \| | Yes | 57 | 27 (23.1) | 0.922 | 1.030 (0.570 –1.862) |
|  | No | 193 | 90 (76.9) |  |  |
| \| **Insulin use** \| \| --- \| | Yes | 147 | 67 (57.3) | 0.644 | 0.888 (0.536 - 1.470) |
|  | No | 103 | 50 (42.7) |  |  |
| \| **Statin use** \| \| --- \| | Yes | 187 | 87 (75.6) | 0.805 | 1.043 (0.749 - 1.452) |
|  | No | 61 | 28 (24.4) |  |  |
| \| **Cardiovascular Disease** \| \| --- \| | Yes | 88 | 52 (44.4) | 0.004 | 2.156 (1.271 – 3.657) |
|  | No | 162 | 65 (55.6) |  |  |
| \| **Hypothyroidism** \| \| --- \| | Yes | 65 | 32 (27.4) | 0.673 | 1.129 (0.641-1.989) |
|  | No | 184 | 85 (72.6) |  |  |
| \| **Diabetic retinopathy** \| \| --- \| | Yes | 93 | 53 (58.2) | 0.003 | 2.406 (1.360 – 4.356) |
|  | No | 107 | 38 (41.8) |  |  |
| \| **Macular edema** \| \| --- \| | Yes | 27 | 17 (19.3) | 0.046 | 2.346 (1.014 – 5.428) |
|  | No | 169 | 71 (80.7) |  |  |
| \| **Diabetic renal disease** \| \| --- \| | Yes | 121 | 57 (55.3) | 0.714 | 1.104 (0.651 – 1.870) |
|  | No | 103 | 46 (44.7) |  |  |
| \| **Diabetic neuropathy** \| \| --- \| | Yes | 40 | 22 (18.8) | 0.281 | 1.454 (0.736 – 2.870) |
|  | No | 208 | 95 (81.2) |  |  |
| \| **GFR < 60 ml/min/1.73 m2** \| \| --- \| | Yes | 61 | 32(28.1) | 0.317 | 1.346 (0.753 – 2.406) |
|  | No | 182 | 82 (71.9) |  |  |
| \| **ACR > 30 mg/g creatinine** \| \| --- \| | Yes | 101 | 45 (45.5) | 0.768 | 0.923 (0.540 - 1.577) |
|  | No | 116 | 54 (54.5) |  |  |
| **Gender** | Female | 109 | 55 (47.0) | 0.285 | 1.314 (0.796 – 2.169) |
|  | Male | 142 | 62 (53.0) |  |  |
| \| **BMI > 30 kg/m2** \| \| --- \| | Yes | 128 | 58 (45.3) | 0.629 | 0.885 (0.538 – 1.455) |
|  | No | 122 | 70 (54.7) |  |  |
| **HBA1c** $\boldsymbol{\geq}$ **7 %** | Yes | 167 | 82 (49.1) | 0.291 | 1.351 (0.773 – 2.360) |
|  | No | 72 | 85 (50.9) |  |  |

P <0.25 = significant to be used in multivariate binary logistic regression model

PHQ-9 = Patient Health Questionnaire, GFR = Glomerular filtration rate, RAC = Albumin/creatinine ratio, BMI = Body mass index, GCS = Global cognitive score (z)

**Table 2: Baseline Multivariate Binary Logistic Regression Model - GCS < 0 and explanatory variables***

| Variable | Classification | N | GCS 1 < 0  n (%) | P | Odds Ratio (CI 95%) |
| --- | --- | --- | --- | --- | --- |
| **Age**  **(years)** | $\geq$ 65 | 106 | 67 (57.3) | 0.000 | 5.461 (2.417– 12.344) |
|  | $<$ 65 | 145 | 50 (42.7) |  |  |
| **School education**  **(years)** | $\leq$ 6 | 166 | 87 (74.4) | 0.000 | 12.192 (5.618 – 26.459) |
|  | $>$ 6 | 133 | 30 (25.6) |  |  |
| **Arterial hypertension** | Yes | 206 | 106 (90.6) | 0.086 | 2.545 (0.877 – 7.389) |
|  | No | 44 | 11 (9.4) |  |  |
| **PHQ-9 s > 9** | Yes | 93 | 50 (42.7) | 0.003 | 3.533 (1.546 – 8.075) |
|  | No | 158 | 67 (57.3) |  |  |
| **Diabetic retinopathy** | Yes | 93 | 53 (58.2) | 0.017 | 2.505 (1.176 – 5.340) |
|  | No | 107 | 38 (41.8) |  |  |

Note: Method Backward conditional

*Variables included: age ≥ 65 years, ≤ 6 school years, Diabetes duration ≥ 10 years, physical activity, PHQ-9 score > 9, arterial hypertension, cardiovascular disease, diabetic retinopathy, macular edema.

GCS = Global cognitive score (z)

**Table 3: Follow up Univariate Binary Logistic Regression Model - GCS < 0 and explanatory variables***

| Variable | Classification | N | GCS follow up < 0  n (%) | P* | Odds Ratio (CI 95%) |
| --- | --- | --- | --- | --- | --- |
| **Age (years)** | $\geq$ 65 | 54 | 28 (50.9) | 0.038 | 2.114(1.043 – 4.286) |
|  | $<$ 65 | 80 | 27 (49.1) |  |  |
| **School education (years)** | ≤ 6 | 75 | 43 (78.2) | 0.000 | 8.725 (3.908 -19.479) |
|  | > 6 | 75 | 12 (21.8) |  |  |
| **Physical activity** | Yes | 41 | 11 (20.0) | 0.029 | 0.408 (0.183 – 0.910) |
|  | No | 93 | 44 (80.0) |  |  |
| **Tabbacco smoker** | Yes | 57 | 23 (41.8) | 0.888 | 0.951 (0.474 – 1.909) |
|  | No | 77 | 32 (58.2) |  |  |
| **Alcoholic** | Yes | 45 | 20 (36.4) | 0.570 | 1.234 (0.597 – 2.550) |
|  | No | 89 | 35 (63.6) |  |  |
| **Diabetes duration (years)** | $\geq$ 10 | 78 | 41 (74.5) | 0.002 | 3.324 (1.569– 7.042) |
|  | $<$ 10 | 56 | 14 (25.5) |  |  |
| **Severe Hypoglycemia** | Yes | 29 | 12 (21.8) | 0.967 | 1.018 (0.442– 2.346) |
|  | No | 105 | 43 (78.2) |  |  |
| **Arterial hypertension** | Yes | 106 | 52 (94.5) | 0.001 | 8.025 (2.284 – 28.196) |
|  | No | 28 | 3 (5.5) |  |  |
| **PHQ-9 > 9** | Yes | 53 | 29 (52.7) | 0.010 | 2.556 (1.251 – 5.221) |
|  | No | 81 | 26 (47.3) |  |  |
| **Depression**  **/Anxiety** | Yes | 24 | 10 (18.2) | 0.946 | 1.032 (0.421 –2.528) |
|  | No | 110 | 45 (81.8) |  |  |
| **Insulin use** | Yes | 96 | 37 (67.3) | 0.290 | 1.475 (0.718 – 3.027) |
|  | No | 54 | 18 (32.7) |  |  |
| **Sstatin use** | Yes | 106 | 46 (85.2) | 0.197 | 1.821 (0.732 – 4.528) |
|  | No | 27 | 8 (14.8) |  |  |
| **Cardiovascular disease** | Yes | 38 | 25 (45.5) | 0.000 | 4.231 (1.906- 9.389) |
|  | No | 96 | 30 (54.5) |  |  |
| **Hypothyroidism** | Yes | 38 | 18 (32.7) | 0.350 | 1.435 (0.673-3.062) |
|  | No | 96 | 37(67.3) |  |  |
| **Diabetic retinopathy** | Yes | 56 | 30 (58.8) | 0.008 | 2.692 (1.294 – 5.603) |
|  | No | 70 | 21 (41.2) |  |  |
|  |  |  |  |  | (continue) |
| **Macular edema** | Yes | 14 | 9 (17.6) | 0.063 | 3.000 (0.942 - 9.553) |
|  | No | 112 | 42 (82.4) |  |  |
| **Diabetic renal disease** | Yes | 62 | 26 (48.1) | 0.930 | 1.032 (0.513 – 2.074) |
|  | No | 68 | 28 (51.9) |  |  |
| **Diabetic neuropathy** | Yes | 26 | 9 (16.4) | 0.459 | 0.714 (0.292 – 1.744) |
|  | No | 108 | 46 (83.6) |  |  |
| **GFR < 60**  **ml/min/1.73m^2^** | Yes | 28 | 15 (27.3) | 0.143 | 1.875 (0.809 – 4.346) |
|  | No | 105 | 40 (72.7) |  |  |
| **ACR**  **> 30 mg/g creatinine** | Yes | 49 | 19 (35.2) | 0.579 | 0.814 (0.394 – 1.681) |
|  | No | 80 | 35 (64.8) |  |  |
| **HBa1c** $\geq$ **7 %** | Yes | 92 | 41 (74.5) | 0.261 | 1.550 (0.721-3.333) |
|  | No | 41 | 14 (25.5) |  |  |
| **Gender** | Female | 70 | 26 (47.3) | 0.338 | 1.402 (0.703 - 2.798) |
|  | Male | 64 | 29 (52.7) |  |  |
| **BMI** $\geq30$  **(kg/m^2^)** | Yes | 69 | 26 (47.3) | 0.415 | 0.751 (0.376 – 1.497) |
|  | No | 65 | 29 (52.7) |  |  |

P <0.25 = significant to be used in multivariate binary logistic regression model

PHQ-9: Patient Health Questionnaire, GFR: Glomerular filtration rate, RAC: Albumin/creatinine ratio, BMI: Body mass index, GCS: Global cognitive score (z)

**Table 4: Follow up Multivariate Binary Logistic Regression Model - GCS < 0 and explanatory variables***

| Variable | Classification | N | GCS follow up < 0  n (%) | P | Odds Ratio (CI 95%) |
| --- | --- | --- | --- | --- | --- |
| Schooling  (years) | ≤ 6 | 66 | 43 (78.2) | 0.000 | 10.154 (3.681 – 28.010) |
|  | > 6 | 68 | 12 (21.8) |  |  |
| Diabetes duration (years) | $\geq$ 10 | 78 | 41 (74.5) | 0.060 | 2.679 (0.959 – 7.484) |
|  | $<$ 10 | 56 | 14 (25.5) |  |  |
| PHQ-9 > 9 | Yes | 53 | 29 (52.7) | 0.002 | 4.918 (1.766 – 13.695) |
|  | No | 81 | 26 (47.3) |  |  |
| Arterial hypertesnion | Yes | 106 | 52 (94.5) | 0.019 | 7.021 (1.381– 35.711) |
|  | No | 28 | 3 (5.5) |  |  |
| Cardiovascular  disease | Yes | 38 | 25 (45.5) | 0.002 | 5.758 (1.930 – 17.183) |
|  | No | 96 | 30 (54.5) |  |  |

*Variables included: age ≥ 65 years, ≤ 6 school years, diabetes duration ≥ 10 years, physical activity, PHQ-9 > 9, arterial hypertension, cardiovascular disease, diabetic retinopathy, macular edema, GCS: Global cognitive score (z)
